# Effect of dual-acupoint and single-acupoint electric stimulation on postoperative outcomes in elderly patients subjected to gastrointestinal surgery: study protocol for a randomized controlled trial

**DOI:** 10.1186/s13063-018-3052-2

**Published:** 2018-12-04

**Authors:** Zhi-hong Lu, Hai-long Dong, Jia-wen Huang-fu, Xiao-jian Fan, Wei-xian Zhao, Su Min, Wei Zhang, Ming-fu Liu, Yong-hui Wang, Li-ni Wang, Li-ze Xiong

**Affiliations:** 1 Department of Anesthesiology, Xijing Hospital, Fourth Military Medical University, Xi’an, 710032 Shaanxi China; 2 Department of Anesthesiology, Second Affiliated Hospital of Guangzhou University of Traditional Chinese Medicine, Guangzhou, China; 3 Department of Anesthesiology, First Affiliated Hospital of Chongqing Medical University, Chongqing, China; 4 Department of Anesthesiology, First Affiliated Hospital of Zhengzhou University, Zhengzhou, China

**Keywords:** Transcutaneous electric acupoint stimulation, Postoperative complication, Elderly patients, Study protocol

## Abstract

**Background:**

Transcutaneous electric acupoint stimulation (TEAS) has shown benefits when used peri-operatively. However, the role of numbers of areas with acupoint stimulation is still unclear. Therefore, we report the protocol of a randomized controlled trial of using TEAS in elderly patients subjected to gastrointestinal surgery, and comparing dual-acupoint and single-acupoint stimulation.

**Methods/design:**

A multicenter, randomized, controlled, three-arm design, large-scale trial is currently undergoing in four hospitals in China. Three hundred and forty-five participants are randomly assigned to three groups in a 1:1:1 ratio, receiving dual-acupoint TEAS, single-acupoint TEAS, and no stimulation, respectively. The primary outcome is incidence of pulmonary complications at 30 days after surgery. The secondary outcomes include the incidence of pulmonary complications at 3 days after surgery; the all-cause mortality within 30 days and 1 year after surgery; admission to the intensive care unit (ICU) and length of ICU stay within 30 days after surgery; the length of postoperative hospital stay; and medical costs during hospitalization after surgery.

**Discussion:**

The result of this trial (which will be available in September 2019) will confirm whether TEAS before and during anesthesia could alleviate the postoperative pulmonary complications after gastrointestinal surgery in elderly patients, and whether dual-acupoint stimulation is more effective than single-acupoint stimulation.

**Trials registrations:**

ClinicalTrials.gov, ID: NCT03230045. Registered on 10 July 2017.

**Electronic supplementary material:**

The online version of this article (10.1186/s13063-018-3052-2) contains supplementary material, which is available to authorized users.

## Background

Life expectancy has been increasing in recent years. In parallel with the increase of life expectancy, the number of elderly patients undergoing surgery is rising. Some of the diseases, such as gastrointestinal cancers, are predominant diseases of this elderly population and the major causes of morbidity and mortality. It had been reported that more than 30% patients with gastric cancer were older than 70 years [1]. Age exceeding 70 years is an independent predictor of increased postoperative complications, in-hospital mortality, and longer hospital stays [2]. Among the postoperative complications, pulmonary complications are closely related to general anesthesia. Atelectasis develops within minutes after the induction of general anesthesia [3] and age is a risk factor of pulmonary complications. Almost all mechanically ventilated, elderly patients developed atelectasis and shunting during anesthesia [4].

Acupuncture is an integral part of an ancient Chinese system of medicine that has been used for more than 2500 years to treat diseases and relieve pain. The use of acupuncture during anesthesia, which is called anesthesia-assisted-anesthesia, is promising in three aspects [5–7]: decreasing the need for anesthetics such as opioids, alleviating adverse events, and exerting organ-protective effects. Electrical stimulation of acupoints, which includes electroacupuncture and transcutaneous electric acupoint stimulation (TEAS), was developed as an alternative to manual acupuncture. Electrical stimulation has several advantages over manual stimulation, such as causing less pain, being less time consuming, and with better standardization.

However, whether the more acupoints stimulated the better the benefit is still in debate. Though according to the Traditional Chinese Medicine (TCM) theory, combinative stimulation of acupoints is important, some investigators found that there was no difference between dual-acupoint and single-acupoint stimulation [8]. In this study, we hypothesize that TEAS before and during surgery would decrease the morbidity or mortality of major postoperative pulmonary complications at 30 days after gastrointestinal surgery in elderly patients, and dual-acupoint stimulation would be better than single-acupoint stimulation.

## Methods/design

### Ethical issues

The sponsor is responsible for the study. The study will be conducted in accordance with the protocol, applicable regulatory requirements and the ethical principles of the Declaration of Helsinki as adopted by the 18th World Medical Assembly in Helsinki, Finland, in 1964 and subsequent versions. The study protocol and statistical analysis plan have been approved for all centers by a central ethics committee (Ethics Committee, Xijing Hospital, Fourth Military Medical University, Xi’an, Shaanxi, China) (No. 20162019-X-1) according to Chinese law. The study has been registered at ClinicalTrials.gov (NCT03230045). All patients should give written informed consent prior to entering the study. The investigator should inform the patients of the protocol, objective and possible risks of the study. And the patients should be assured that they could quit the study any time.

### Design

**Fig. 1:**
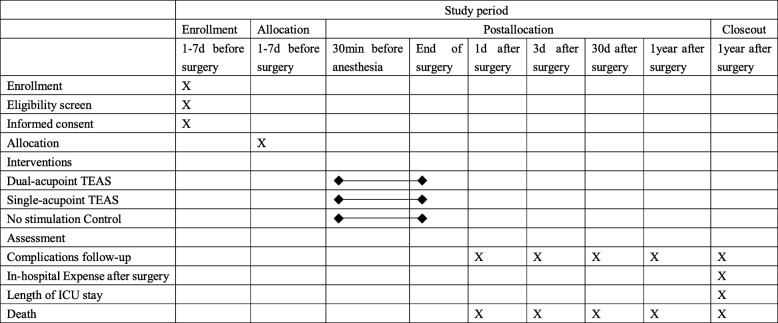
Standard Protocol Items: Recommendations for Interventional Trials (SPIRIT) Figure. Interventions and assessments will be administered at different time points (indicated by X). See description within the manuscript for more details. *TEAS* transcutaneous electric acupoint stimulation

We adhered to the Standard Protocol Items: Recommendations for Interventional Trials (SPIRIT) guidelines in the preparation of this protocol (see Fig. 1 and Additional file 1 for the SPIRIT Figure and Checklist, respectively). The trial is an investigator-initiated, multicenter, randomized clinical trial comparing treatment with dual-acupoint TEAS versus single-acupoint stimulation and no-stimulation before and during anesthesia in elderly patients undergoing gastrointestinal surgery. Three hundred and forty-five participants will be included from the following four hospitals: First Affiliated Hospital of Fourth Military Medical University (Xijing Hospital); Second Affiliated Hospital of Guangzhou University of TCM; First Affiliated Hospital of Chongqing Medical University; and First Affiliated Hospital of Zhengzhou University. These participants will be randomly assigned to three groups through central randomization in a 1:1:1 ratio. The central randomization system will be used and performed by the Department of Anesthesiology of Xijing Hospital. In each center, an investigator who is not involved in anesthesia and follow-up will perform the randomization. This investigator logs into the central randomization system and inputs the patient’s information, then a random number and group assignment will be immediately given by the system. According to the assignment, the patients will receive interventions from 30 min before anesthesia induction to the end of the surgery. The follow-up period lasts for 1 year. Outcomes are assessed at baseline and 1, 3, and 30 days, and 1 year after surgery.

### Patients

#### Study population

The study focuses on patients aged 65 years or older who were scheduled for elective gastrointestinal surgery under general anesthesia.

#### Inclusion criteria

Participants will be included if they fulfill the following conditions: scheduled for elective gastrointestinal surgery under general anesthesia; aged 65 years or older; provided a signed written consent form.

#### Exclusion criteria

Patients with any of the following conditions will be excluded: contraindications to the use of electroacupuncture (including those with infection or injury of the skin to attach electrodes, and those with implanted electronic devices); difficulty in communication; surgery within 12 h of admission to hospital; preoperative pneumonia or on ventilator; history of lung surgery; surgery involving thoracic manipulation; being involved in other clinical trials in the last 3 months.

### Interventions and comparison

#### Rationale for acupuncture protocol

On the day before surgery, patients are randomly assigned to the dual-acupoint TEAS group (DT), the single-acupoint TEAS group (ST) or the Control group, stratified by study center with a central computer-generated scheme. For all the patients, the electrodes (5 × 5 mm^2^) are placed at bilateral ST36 (*Zusanli*) and BL13 (*Feishu*), and then connected to the Hwato Electric Acupuncture Treatment Instrument (model No. SDZ-V; Suzhou Medical Appliances Co, Ltd., Suzhou, China). Thirty minutes before surgery, the intervention is performed by an investigator who is not involved in anesthesia and follow-up. For group DT, ST36 and BL13 are stimulated. For group ST, only ST36 is stimulated. For the Control group, electrodes are attached but no stimulation is given.

ST36 and BL13 are chosen and identified according to ancient Chinese medical books. ST36 is the most often used acupoint for abdominal surgery, and BL13 may benefit the respiratory system. ST36 is located on the anterior aspect of the leg, at 3 *cun* (Chinese inch) below the kneecap and 1 *cun* from the anterior crest of the tibia. BL13 is located at 1.5 *cun* lateral to the spinous process of the third thoracic vertebrae. One *cun* is defined as the width of the patient’s thumb (see supplementary material 2). The patients in the DT and ST groups receive electrical stimulation with the “disperse-dense” waves of alternating frequencies of 2 and 15 Hz for a 2-s cycle. Increasing electrical stimulation intensity (7–11 mA) will be applied to identify the threshold intensity, defined as perception of the “*Teh Chi*” sensations of heaviness, numbness, and swelling in their crura. Electric stimulation is administered 30 min prior to surgery and continued until the end of the surgery. Patients in the Control group receive the same treatment without electric stimulation.

#### Blinding

All patients will never have been subjected to transcutaneous electrical stimulation treatment before. Patients were told that they may or may not be able to feel the electrical stimulation. The skin surface was sterilized by alcohol and electrodes were pasted to bilateral ST36 and BL13. The generator was connected by leads to two sterile electrodes with a pre-gelled contact surface (approximately 1.5 in. × 1.5 in.) by a specific investigator who is not involved in anesthesia and follow-up. Treatment assignments were concealed from patients, data collector, research staff, the statistician, and the Data and Safety Monitoring Committee. The stimulator is placed in an opaque box to keep the surgical team and the anesthetists blinded to group allocation. The patients and the staff on the wards are not informed during follow-up about the intervention received. An independent Data and Safety Monitoring Committee oversees the study conduct and reviews the blinded safety data. Unmasking will be done when a severe adverse event happens. The Committee consists of a surgeon, an intensive care unit (ICU) physician and an anesthesiologist who is not involved in the study. The members of the Steering Committee vouch for the accuracy and completeness of the data and analyses and the fidelity of the study to the protocol.

At the end of the follow-up, to evaluate participant blinding, the patients are asked which of the groups they believed they belonged to and to give a reason for their choice. No investigator has access to patient allocation and outcome data at the same time. All statistical analyses are performed with the intervention groups named A, B, and C, and the statistician is blinded to allocation.

### Operative details

All surgeries will be done under general anesthesia with tracheal intubation. Anesthesiologists monitor and record the following parameters during surgery: electrocardiogram (ECG), heart rate (HR), blood pressure (BP), respiratory rate (RR), oxygen saturation (SpO_2_), end-tidal CO_2_ (EtCO_2_), temperature, urine volume, and blood gases. Protective lung ventilation with a tidal volume of 6 to 8 ml/kg of predicted body weight, a PEEP of 6 to 8 cmH_2_O will be used. Postoperatively, patients will be transferred to the ward or ICU depending on their condition. Intravenously administered, patient-controlled analgesia with sufentanil will be used for postoperative analgesia.

At the end of the surgery, the information is recorded according to the Checklist. Symptoms are recorded. All chest radiographs and computed tomograms (CTs) are evaluated for infiltrates and atelectasis by the attending radiologist, who is unaware of the intervention applied.

The investigators enter all data into a central database for the trial. A summary of each patient data record is forwarded to the principle investigator of each center 30 days after surgery for inspection. When follow-up finishes, the patient records are transmitted to the statistical coordinating center for editing and analysis.

Patients are excluded from the protocol analysis if they did not meet the inclusion criteria, fulfilled an exclusion criterion, had no in-hospital evaluation of the outcomes or had no follow-up visit.

### Outcome measurement

*The primary outcome* is postoperative pulmonary complications occurring within 30 days after surgery.

Postoperative pulmonary events are scored on an ordinal scale of 1 to 4 (see supplementary material 3). A clinically significant postoperative pulmonary complication is defined as two or more items in the grade-2 complications or one item in the grade-3 or grade-4 complications.

*Secondary outcomes* include the incidence of postoperative pulmonary complications within 30 days after surgery; all-cause mortality within 30 days and 1 year after surgery; admission to the intensive care unit (ICU) and length of ICU stay within 30 days after surgery; the length of postoperative hospital stay; and medical costs during hospitalization after surgery.

### Sample size calculation and statistical analysis

To calculate sample size, we used available data from the pilot study in our hospital on postoperative pulmonary complication incidence (unpublished). In the study, the incidence of postoperative pulmonary complications was 23% in the ST-36 TEAS group and 32% in the Control. Assuming an additional decrease by 9% in group DT (14%), with a power of 80% at the *P* = 0.05 level of statistical significance, 345 patients (*N* = 115 for each group) are needed.

Statistical analysis will be performed by the Department of Statistics of the Fourth Military Medical University. The statistician is blinded from the allocation of groups. SPSS19.0 statistical software packages will be used to analyze the data. Prior to all analyses, a detailed statistical analysis protocol was developed. The intention-to-treat (ITT) population was defined as the patients who are randomized and received at least one treatment session. The per-protocol (PP) population was defined as the patients who completed the study and did not have major protocol violations. All analyses were based on the ITT population and the PP population. The result of the ITT analysis will be compared with that of the PP analysis to check whether the results are consistent.

All data will be checked by two investigators. We compare postoperative variables with either Student’s *t* test or the Mann-Whitney *U* test for continuous variables, depending on the characteristics of the variables, and we use the *χ*^2^ test for categorical variables. We compare both the composite primary outcome of incidence of postoperative pulmonary complications within 30 days after surgery and the secondary outcome of occurrence of systemic inflammatory response syndrome (SIRS) complications by postoperative day 30 with an unadjusted *χ*^2^ test, weighting every individual complication equally. Comparison between the groups is made by analyzing data with the post-hoc method. Where appropriate, we express statistical uncertainty with 95% confidence intervals (CIs). We calculate Kaplan-Meier estimates of survival curves, and we use log-rank tests to compare survival distributions between study groups. We will censor data used for Kaplan-Meier estimates when patients do not have a postoperative pulmonary complication during the study period. Analyses are performed using SPSS 13.0 software and a two-sided *P* < 0.05 was considered statistically significant.

Interim analyses are performed after half the required participants had been enrolled (*n* = 173). If the Institutional Review Board thought it was no longer ethical to withhold TEAS from the patients in the non-acupoint and Control groups based on the results of the interim analysis with pulmonary complications as the endpoint, the study will be stopped.

## Discussion

The result of this trial is expected to provide convincing evidence that TEAS is effective for improving postoperative outcomes in older patients undergoing gastrointestinal surgery, and that dual-acupoint TEAS is better than single-acupoint.

TEAS is an alternative technique to acupuncture. Compared with acupuncture, it is easier and safer for non-acupuncturists to use. The use of acupuncture-related techniques during anesthesia may benefit patients considerably, especially by decreasing anesthetic consumption and reducing adverse events [6–11]. We have used electroacupuncture in clinical settings and obtained positive results. In healthy volunteers, we proved that electroacupuncture could alleviate low-perfusion-induced cerebral dysfunction [12]. In both adult and pediatric patients undergoing cardiac surgery, we proved that TEAS could improve cardiac outcomes and shorten ICU stays [13, 14]. Based on these evidences, we speculated that TEAS pretreatment would activate the endogenous protective mechanism, and then protect patients against subsequent surgical stress. We believe that this intervention will reduce the subsequent incidence, duration, and severity of organ dysfunction, and further reduce the morbidity and mortality. However, whether the more acupoints stimulated the better the benefit is still not clear. According to the TCM theory, combinative stimulation of acupoints is important. But Alizadeh et al. found that there was no difference between dual-acupoint and single-acupoint stimulation for treating postoperative nausea and vomiting [8].

However, currently there is not enough evidence for acupuncture based on strict clinical trial of evidence-based medicine because of the poor quality of current studies, such as small sample size, no description of methods for randomization, no standardized acupuncture protocol which may lead to performance bias, and so on. In this trial, we used a central randomization, multicenter design, standardized acupuncture protocol, with a large sample size to ensure power. A major issue in planning the design of this trial is which control should be used. Since the primary consideration of this trial is to clarify whether TEAS is effective in reducing postoperative complications, we all agree that both a non-acupoint Control group and a no-stimulation Control group are needed.

We focus on a specific population though this trial is a pragmatic design. First, we exclude patients with severe comorbidities because, in these patients, the postoperative complications could be so severe that protection by TEAS will be too weak to be observed. Second, we exclude patients with contraindications to electrical stimulation, to ensure that TEAS would not do harm to these patients.

Based on TCM theory, the onset of acupuncture’s effect is 15–30 min, and acupuncture during anesthesia may be not comparable to that in conscious patients. Previous studies in which acupuncture was started after the induction of anesthesia revealed reduced efficacy or a lack of efficacy, although the mechanism underlying this phenomenon is not known [15, 16]. So in our study we will start the TEAS at 30 min before anesthesia induction.

Considering that among the postoperative complications, pulmonary complications are closely related to general anesthesia, we choose the incidence of pulmonary complications as primary outcome. Atelectasis develops within minutes after the induction of general anesthesia [4] and age is a risk factor for pulmonary complications. Age-related changes in pulmonary function [17] may increase the possibility of intraoperative lung injury. Almost all mechanically ventilated elderly patients developed atelectasis and shunt during anesthesia [5].

A limitation of this study is the validity of blinding, since interventions will begin when the participants are awake. To solve the problem, for all participants, we attached electrodes to all acupoints and the patients are told that they may or may not feel the stimulation. At the end of the follow-up, to evaluate participant blinding, the patients also are asked which of the groups they believed they belonged to and to give a reason for their choice. By these manipulations, we hope to blind the participants.

In conclusion, the results of this trial are expected to confirm whether this acupuncture-related technique is effective in decreasing postoperative complications in elderly patients.

### Trial status

The first participants were included on 8 October 2017, and this article was submitted on 6 March 2018. To date, 113 participants have been recruited.

## Additional file

Additional file 1: Standard Protocol Items: Recommendations for Interventional Trials (SPIRIT) 2013 Checklist: recommended items to address in a clinical trial protocol and related documents. Measurement of “*Cun*,” locations of ST36 and BL13. Scale of postoperative pulmonary complications. (DOCX 1015 kb)

## Abbreviations

ICU: Intensive care unit
ITT: Intention-to-treat
PP: Per-protocol
SPIRIT: Standard Protocol Items: Recommendations for Interventional Trials
TCM: Traditional Chinese medicine
TEAS: Transcutaneous electric acupoint stimulation
